# The ceRNA PVT1 inhibits proliferation of ccRCC cells by sponging miR-328-3p to elevate FAM193B expression

**DOI:** 10.18632/aging.203514

**Published:** 2021-09-13

**Authors:** Guohai Xie, Xinyi Zheng, Zhong Zheng, Ruoyu Wu, Zhixian Yao, Wenjie Huang, Feng Sun, Xingyu Mu, Ke Wu, Junhua Zheng

**Affiliations:** 1 Department of Urology, Ningbo First Hospital, Ningbo 315000, Zhejiang, China; 2 Tongji University School of Medicine, Shanghai 20092, Shanghai, China; 3 Ningbo Clinical Research Center for Urological Disease, Ningbo First Hospital, Ningbo 315010, Zhejiang, China; 4 Department of Pharmacy, Huashan Hospital, Shanghai 200040, Shanghai, China; 5 Department of Urology, Shanghai General Hospital, Shanghai 200080, Shanghai, China

**Keywords:** PVT1, proliferation, clear cell renal cell carcinoma, miR-328-3p, FAM193B

## Abstract

Clear cell renal cell carcinoma (ccRCC) is a common and fatal malignancy. Long noncoding RNAs (lncRNAs) have emerged as crucial biomarkers and regulators in many cancers, warranting the detailed investigation of their biological functions and molecular mechanisms. In this study, we explored the role and mechanism of plasmacytoma variant translocation 1 (PVT1), a competitive endogenous RNA (ceRNA) in ccRCC tissues *in vitro* and *in vivo*. We found that PVT1 is upregulated in ccRCC cells and promoted cell proliferation. Bioinformatic analysis, dual-luciferase reporter assays, argonaute 2-RNA immunoprecipitation (AGO2-RIP), quantitative PCR arrays, western blot assay, and rescue experiments were conducted to explore the underlying mechanisms of PVT1. Our analyses revealed that miR-328-3p was a direct target of PVT1 and that FAM193B was a direct target of miR-328-3p. FAM193B is upregulated in ccRCC tissues and promotes cell proliferation by activating the MAPK/ERK and PI3K/AKT pathways. Our results indicated that PVT1 promotes ccRCC cells proliferation by sponging miR-328-3p to upregulate FAM193B and activate the MAPK/ERK and PI3K/AKT pathways. Collectively, these results suggest that PVT1- miR-328-3p-FAM193B loop could serve as a potential biomarker and therapeutic target for ccRCC.

## INTRODUCTION

Renal cell malignancy or carcinoma (RCC) accounts for the sixth and tenth most frequently diagnosed malignant neoplasms in men and women, respectively, of which approximately 70–80% of cases are clear cell RCC (ccRCC) [1]. Moreover, the survival of patients with metastasis is only 30%, despite the significant advances in cancer diagnosis and therapy [2]. Hence, it constitutes a serious public health concern and the discovery of novel clinically applicable biomarkers as well as effective therapeutic targets for the early detection and appropriate management of underlying patient subtypes, is critically needed.

Long non-coding RNAs (lncRNAs), previously believed to be the noise of transcriptome, are a cluster of RNA fragments longer than 200 nucleotides. However, recently, scientists have uncovered their diverse biological functions, including regulation of telomere length, meiotic entry, retrotransposon silencing, pluripotency, cell cycle coordination, dosage compensation, and imprinting [3]. Moreover, numerous studies have implicated various lncRNAs in various cancers, including ccRCC. Despite these reports, most lncRNAs remain uncharacterized.

As an oncogenic lncRNA, plasmacytoma variant translocation 1 (PVT1) was initially reported as a recurrent breakpoint in Burkitt's lymphoma [4]. This cytoplasmic gene sites in chromosome 6;15 translocations of mouse and t (2;8) of human [5]. However, accumulating evidence supports the notion that PVT1 possesses oncogenic functions in various cancers, including those of the digestive system, breast cancer, cervical cancer, and prostate cancer [6–9]. With respect to RCC, Huang reported that in von Hippel–Lindau (VHL)-defective RCC, activation of the hypoxia-inducible transcription factors (HIF) pathway upregulates PVT1 [10]. Further, a retrospective analysis revealed that PVT1 upregulation resulted in lower overall-survival (OS) and disease-free survival (DFS) of ccRCC sufferers [11]. Ren and his colleagues also provided evidence that PVT1 facilitates the proliferation and invasiveness of kidney cancer cells, along with their epithelial-mesenchymal transition (EMT), by negatively regulating miR-16-5p [12]. These studies suggest that PVT1 is involved in tumor formation and cancer development, however, the precise oncogenic mechanism employed by PVT1 in RCC remains unknown.

Herein, we investigated the role of PVT1 in ccRCC tissues. Our bioinformatic analysis and *ex vivo* study revealed that PVT1 is significantly upregulated in ccRCC tissues. We also elucidated its role in tumor development by sponging miR-328-3p (22 nucleotides long cancer related miRNA) and thereby upregulating the PI3K/AKT and MAPK/ERK signaling pathways via Family with sequence similarity 193 member B (FAM193B). FAM193B is encoded by the FAM193B gene, which located on locus p16.3 of chromosome 4. We believe that along with current clinical factors, the PVT1/ miR-328-3p/ FAM193B feedback loop may provide a useful tool for predicting OS and could eventually become a useful diagnostic medical biomarker as well as a potential therapeutic target for RCC.

## RESULTS

### PVT1 is upregulated in ccRCC tissues and PVT1 silencing inhibits ccRCC progression

**Figure 1:**
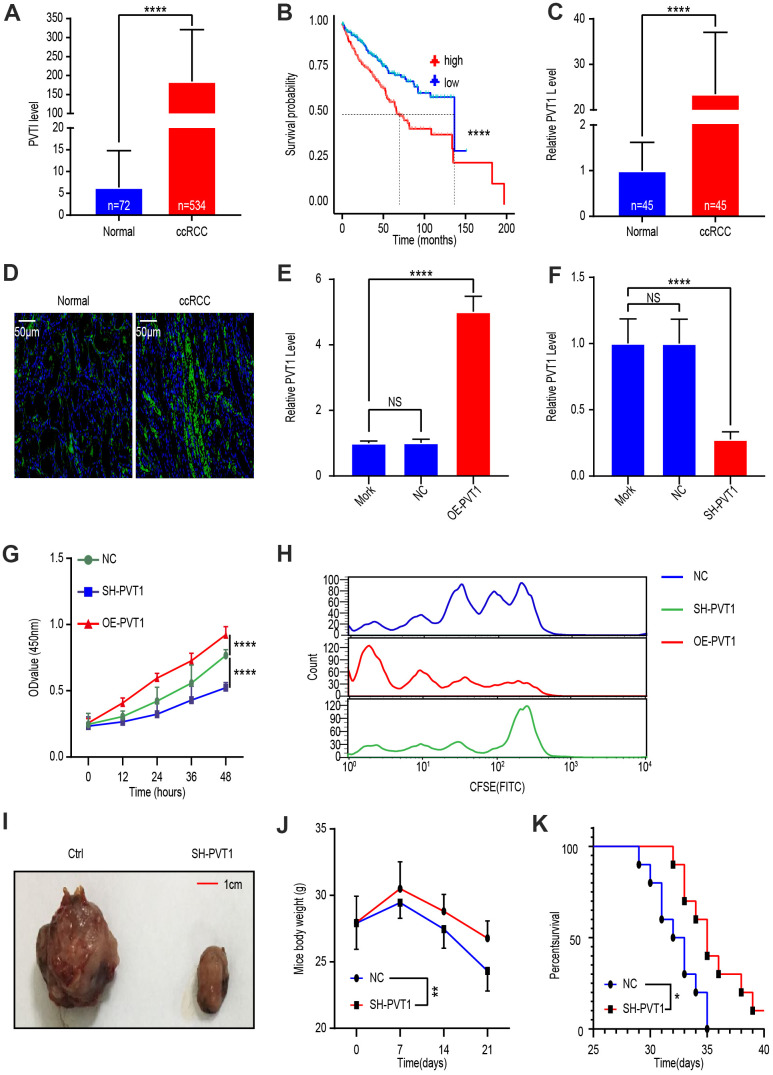
**LncRNA PVT1 is upregulated in ccRCC tissues and promotes cancer cell proliferation.** (**A**) PVT1 expression level in ccRCC compared with that in normal renal tissues according to TCGA database. (**B**) Survival time of ccRCC patients with high or low PVT1 levels according to TCGA database. (**C**) PVT1 levels in ccRCC tissues and adjacent normal renal tissues were determined by qRT-PCR. (**D**) Representative images of fluorescently labeled PVT1 in ccRCC tissues and adjacent normal renal tissues. (**E**) PVT1 expression in Caki-1 cells treated with Mork, Lentivirus vector, or oe-PVT1 Lentivirus, determined by qRT-PCR. (**F**) PVT1 expression in Caki-1 cells treated with Mork, Lentivirus vector or sh-PVT1 Lentivirus, determined by qRT-PCR. (**G**) Cell viability was determined by CCK-8 assays after Lentivirus vector, sh-PVT1 Lentivirus, or oe-PVT1 Lentivirus treatment. (**H**) Fluorescence attenuation in CFSE-labeled-Caki-1 cells after lentivirus vector, SH-PVT1 Lentivirus, or OE-PVT1 lentivirus treatment for 48 hours. (**I**) Tumor shape and size of ccRCC xenograft mouse models harboring tumors generated by cells treated with lentivirus vector or OE-PVT1 lentivirus. (**J**) Body weight of xenograft models harboring tumor generated by cells treated with Lentivirus vector or sh-PVT1 Lentivirus. (**K**) Survival time of xenograft models harboring tumors generated by cells treated with lentivirus vector or SH-PVT1 lentivirus. Mean ± SEM, *P < 0.05, **P < 0.01, ****P < 0.001, NS: no significance.

To investigate PVT1 levels in ccRCC, we analyzed TCGA data of 72 normal kidney tissues and 534 ccRCC tissues. Bioinformatic analysis revealed that PVT1, compared to normal renal tissues, was more highly expressed in the tissues of ccRCC (Figure 1A). We then comparted ccRCC patients into PVT1-low and PVT1-high groups according to TCGA database and found that the survival time of patients was shorter in the former than the latter group (Figure 1B). To verify these analytic results, we obtained 45 ccRCC tissues and those in adjacent normal part from patients at the Shanghai General Hospital. qRT-PCR analysis revealed that PVT1 levels in ccRCC were statistically higher comparing to the adjacent normal part (Figure 1C). These results were confirmed by laser confocal scanning microscopy of fluorescently labeled PVT1 (Figure 1D). Next, to elucidate the function of PVT1 in ccRCC, we exploited the knockdown and overexpression efficiency 24 hours after treatment with either a Mock, lentivirus vector, SH-PVT1 lentivirus, or OE-PVT1 lentivirus. qRT-PCR results confirmed that SH-PVT1 lentivirus effectively reduced PVT1 levels, while OE-PVT1 lentivirus effectively increased PVT1 levels in Caki-1 cells (Figure 1E, 1F). Furthermore, CCK-8 analysis showed PVT1 knockdown significantly hindered Caki-1 cell proliferation, while PVT1 overexpression had the opposite effect (Figure 1G). Flow cytometry analysis demonstrated that following treatment of Caki-1 cells with CFSE-labeled lentivirus vector, SH-PVT1 lentivirus, or OE-PVT1 lentivirus, the fluorescence intensity of the SH-PVT1 lentivirus-treated cells decayed slowly while that of OE-PVT1 lentivirus-treated cells decayed rapidly (Figure 1H). Finally, *in vivo*, a ccRCC xenograft mouse model was established using subcutaneous injection of lentivirus vector- or SH-PVT1 lentivirus-treated Caki-1 cells. The SH-PVT1 lentivirus-treated group exhibited decreased tumor volume and weight loss rate, and increased survival time (Figure 1I–1K). These results indicated that PVT1 upregulated the cancer-promoting lncRNA in ccRCC.

### FAM193B is regulated by PVT1 and FAM193B silencing inhibits ccRCC proliferation

**Figure 2:**
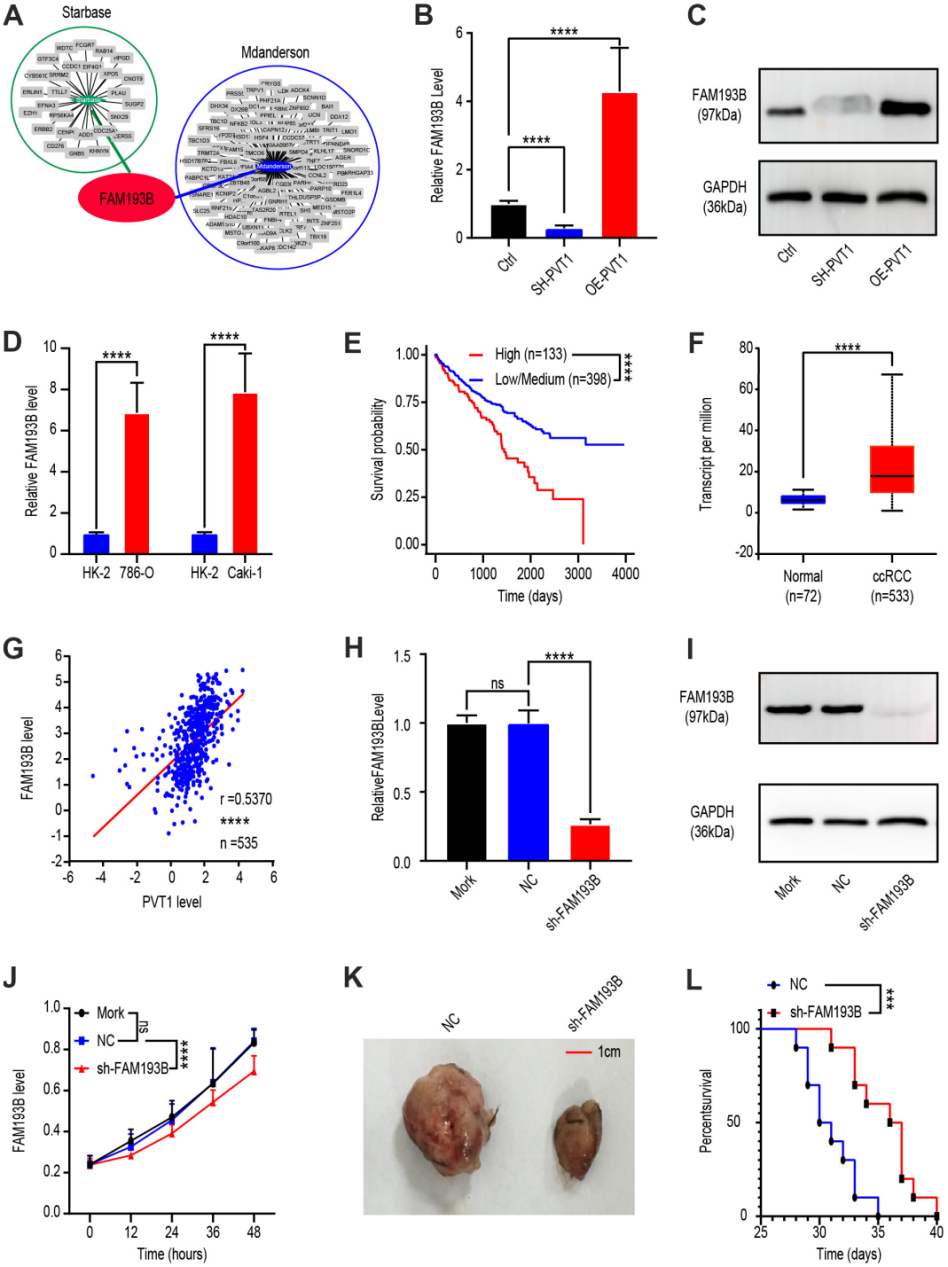
**Effect of PVT1 target gene, FAM193B, on ccRCC cell proliferation *in vitro* and *in vivo*.** (**A**) Potential target gene of PVT1 was calculated and overlapped according to Starbase and MD Anderson databases. (**B**) FAM193B levels were determined by qRT-PCR after treatment with lentivirus vector, SH-PVT1 lentivirus or OE-PVT1 lentivirus for 24 hours. (**C**) Protein levels of FAM193B were determined by western blot after treatment with lentivirus vector, SH-PVT1 Lentivirus, or OE-PVT1 Lentivirus for 48 hours. (**D**) Relative FAM193B levels in 786-O and Caki-1 cells compared to HK-2 cells. (**E**) ccRCC patients were divided into high FAM193B (n=398) and low FAM193B group (n=133) and survival times were recorded according to TCGA database. (**F**) Transcript levels of FAM193B in normal renal tissues (n=72) and ccRCC patients (n=533) according to TCGA database. (**G**) Interrelationship of PVT1 and FAM193B in ccRCC tissues (n=535) according to TCGA database. (**H**) FAM193B levels were determined by qRT-PCR after treatment with Mork, Lentivirus vector or SH-FAM193B Lentivirus for 24 hours. (**I**) Protein levels of FAM193B were determined by western blot after treatment with Mork, Lentivirus vector, or SH-FAM193B lentivirus for 48 hours. (**J**) Cell viability was determined by CCK-8 assay after treatment with Mork, Lentivirus vector, or SH-FAM193B lentivirus and recorded every 12 hours. (**K**) Tumor shape and size of ccRCC xenograft models harboring tumors generated by cells treated with Mork, Lentivirus vector, or SH-FAM193B lentivirus for 30 days. (**L**) Survival time of ccRCC xenograft models harboring tumors generated by cells treated with Mork. Mean ± SEM, ***P < 0.005, ****P < 0.001, NS: no significance.

To explore the relationship between FAM193B and PVT1, we identified potential downstream genes of PVT1 by analyzing the databases, Starbase (http://starbase.sysu.edu.cn/) and MD Anderson (http://www3.mdanderson.org/library/databases/). Both showed that FAM193B was regulated by PVT1 (Figure 2A). To verify this prediction, we treated Caki-1 cells with OE-PVT1 lentivirus, SH-PVT1 lentivirus, and lentivirus empty vector. Western blot analysis and qRT-PCR were then utilized to determine the protein and mRNA levels of FAM193B following transfection for 24 or 48 hours, respectively. The results analytically illustrated that both protein and mRNA levels of FAM193B were positively correlated with PVT1 (Figure 2B, 2C). Moreover, qRT-PCR analysis determined that a significantly higher level of FAM193B was expressed in Caki-1 cell line and 786-O cell line comparing to HK-2 cell line (Figure 2D).

Next, as PVT1 is a carcinogenic non-coding RNA, we sought to determine whether FAM193B promotes ccRCC progression. To this end, we comparted patients with ccRCC into high FAM193B (n = 133) and low/medium FAM193B (n = 398) groups according to TCGA database. Kaplan-Meier survival analytic algorithm showed that, with higher FAM193B levels, patients possessed worse OS than those with low/medium levels (Figure 2E). To verify this positive correlation, we analyzed FAM193B levels in 533 ccRCC patients and compared the results with those of 72 normal renal tissues. Comparing to the normal tissues, FAM193B expression levels were significantly higher in malignant tissues (Figure 2F). Next, we investigated the correlation between FAM193B and PVT1 expression in 533 ccRCC tissues and also observed a positive correlation between FAM193B and PVT1 (Figure 2G). These results demonstrate that FAM193B is positively regulated by PVT1.

To determine whether FAM193B serves as a malignancy promoter in ccRCC cells, we transfected Caki-1 cell line with lentivirus empty vector or SH-FAM193B lentivirus. Western blot and qRT-PCR analyses revealed that SH-FAM193B lentivirus statistically down-regulated FAM193B in Caki-1 cells (Figure 2H, 2I). We subsequently ran a CCK-8 assay and uncovered that cell proliferation was significantly reduced following downregulation of FAM193B (Figure 2J). To identify whether FAM193B influences neoplasm growth *in vivo*, Caki-1 cell line were stably transfected with SH-FAM193B or control vector lentivirus and engrafted subcutaneously into immunodeficient nude mice. Subsequently, all animals developed neoplasms at the site of injection. Nonetheless, the mean volume of malignancies formed by SH-FAM193B–expressing cell line was statistically smaller comparing to the tumors in control group (Figure 2K). Moreover, the survival time of SH-FAM193B–expressing tumor-bearing mice was longer comparing to the group of control (Figure 2L). Collectively, above results confirm the regulation of FAM193B by PVT1, as well as the oncogenic activity of FAM193B in ccRCC, not only *in vitro* yet *in vivo*.

### Latent target miRNAs between PVT1 and FAM193B

**Figure 3:**
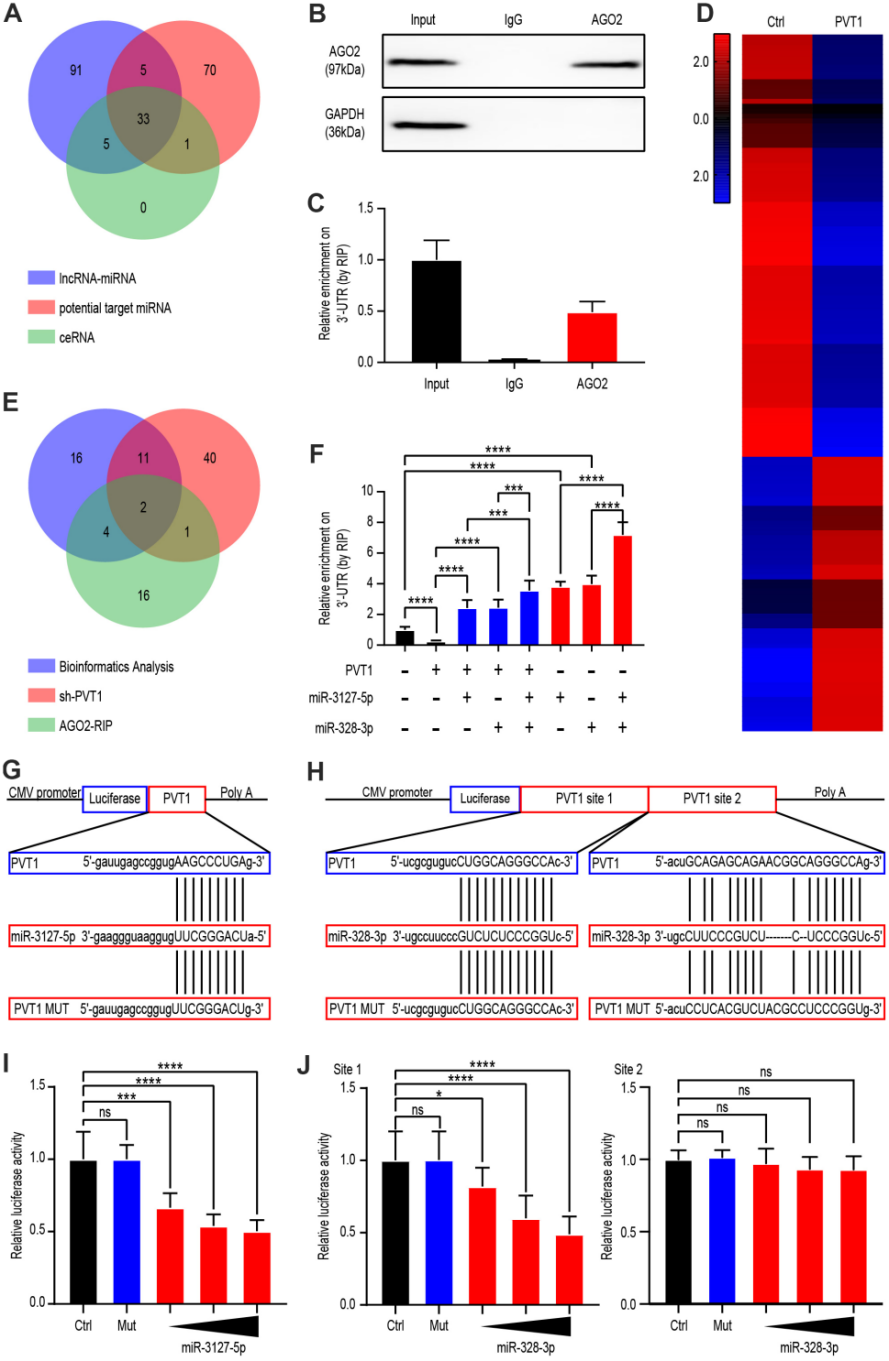
**PVT1 regulated miR-3127-5p and miR-328-3p by serving as a sponge gene.** (**A**) Venn diagram showing the over-lapping of potential target miRNAs of PVT1 according to Starbase database. (**B**) Co-IP and western blot assays indicating the interaction between AGO2 and HuR in AGS cells. (**C**) RIP and qRT-PCR assays revealing the endogenous binding of AGO2 to the 3’-UTR of target genes in Caki-1 cells. (**D**) Heatmaps showing 142 differentially expressed genes (fold change > 2.0, P < 0.05) in Caki-1 cells stably transfected with lentivirus vector or SH-PVT1 lentivirus for 24 hours. (**E**) Venn diagram showing the over-lapping of RNA-seq results from Caki-1 cells stably transfected with sh-PVT1 lentivirus, RIP assays, and potential target miRNAs from Figure 2A. (**F**) RIP and qRT-PCR assays revealing the endogenous binding 3’-UTR of target genes in Caki-1 cells transfected with Mock, PVT1, or miRNA mimics (100 nmol/L). (**G**) Schematic view of miR-3127-5p putative targeting site in the WT and MUT 3’UTR of PVT1. (**H**) Schematic view of miR-328-3p putative target site in the WT and MUT 3’UTR of PVT1. (**I**) Luciferase activity assay in 293 T cells transfected with luciferase reporter plasmids harboring PVT1 3’UTR (WT or MUT) and control miRNA or miR-3127-5p. (**J**) Luciferase activity assay in 293 T cells transfected with luciferase reporter plasmids harboring PVT1 3’UTR (WT or MUT) and control miRNA or miR-328-3p. Mean ± SEM, ***P < 0.005, ****P < 0.001.

Since previous studies suggest that PVT1 affects the miRNA function, we next explored the impacts of PVT1 on miRNA-mediated gene silencing in ccRCC cells. Bioinformatic analysis demonstrated interactions with potential miRNAs regulated by PVT1, FAM193B-related miRNAs, as well as potential ceRNA relationships between PVT1 and FAM193B (Figure 3A). miRNA ribonucleoprotein complexes (miRNPs), which are shown in anti-AGO2 immunoprecipitates, formed RNA-induced silencing complexes (RISCs). Thus, immunoprecipitates by anti-Ago2 include miRNAs and relevant interacting components of RNA. To further explore the regulatory mechanism, argonaute 2-RNA immunoprecipitation system (AGO2-RIP) was used to detect RISCs in ccRCC cells. A RIP assay of the Caki-1 extract was run by utilizing anti-AGO2, the results for which revealed that the enrichment of PVT1 was significantly preferentially in miRNPs with AGO2 comparing to those in immunoprecipitates by anti-IgG (Figure 3B, 3C). Next, Caki-1 cell line were implemented with SH-PVT1 lentivirus for 24 hours and transcriptome sequencing was used to detect differential miRNAs. Heat map generation identified 56 miRNAs as upregulated and 86 miRNAs as down-regulated following PVT1 silencing (P < 0.05, fold change > 2.0) (Figure 3D). Combining the results of previous bioinformatic analysis, AGO2-RIP assays, and miRNA sequencing, we identified miR-3127-5p and miR-328-3p as candidate miRNAs (Figure 3E). Knockdown of PVT1 decreased the endogenous enrichment of PVT1 at the 3′-UTR of target genes; whereas miR-3127-5p and miR-328-3p overexpression had the opposite effect (Figure 3F).

Furthermore, the target nucleotide sequence for recognition of PVT1 was investigated through bioinformatics, and miR-3127-5p and miR-328-3p (site 1 and site 2) were found to have complementary sequences (Figure 3H, 3G). To validate this finding, PVT1 complementary DNA (cDNA) was disposed into the luciferase gene as PVT1-wildetype (PVT-wt) and the co-transfection was done with miR-3127-5p, miR-328-3p (site 1 and site 2) or miR-NC. The activity of luciferase in the miR-3127-5p and miR-328-3p (site 1) group was statistically decreased comparing to the miR-NC group. Further, the miRNA binding site underwent mutation to generate a PVT1-MUT vector. These results demonstrated that the mutated vector posed no significant impingement on the activity of luciferase (Figure 3I, 3J). Overall, these findings indicate that PVT1-miRNA complexes are formed in ccRCC cells.

### PVT1 promotes ccRCC proliferation mainly through miR-328-3p

**Figure 4:**
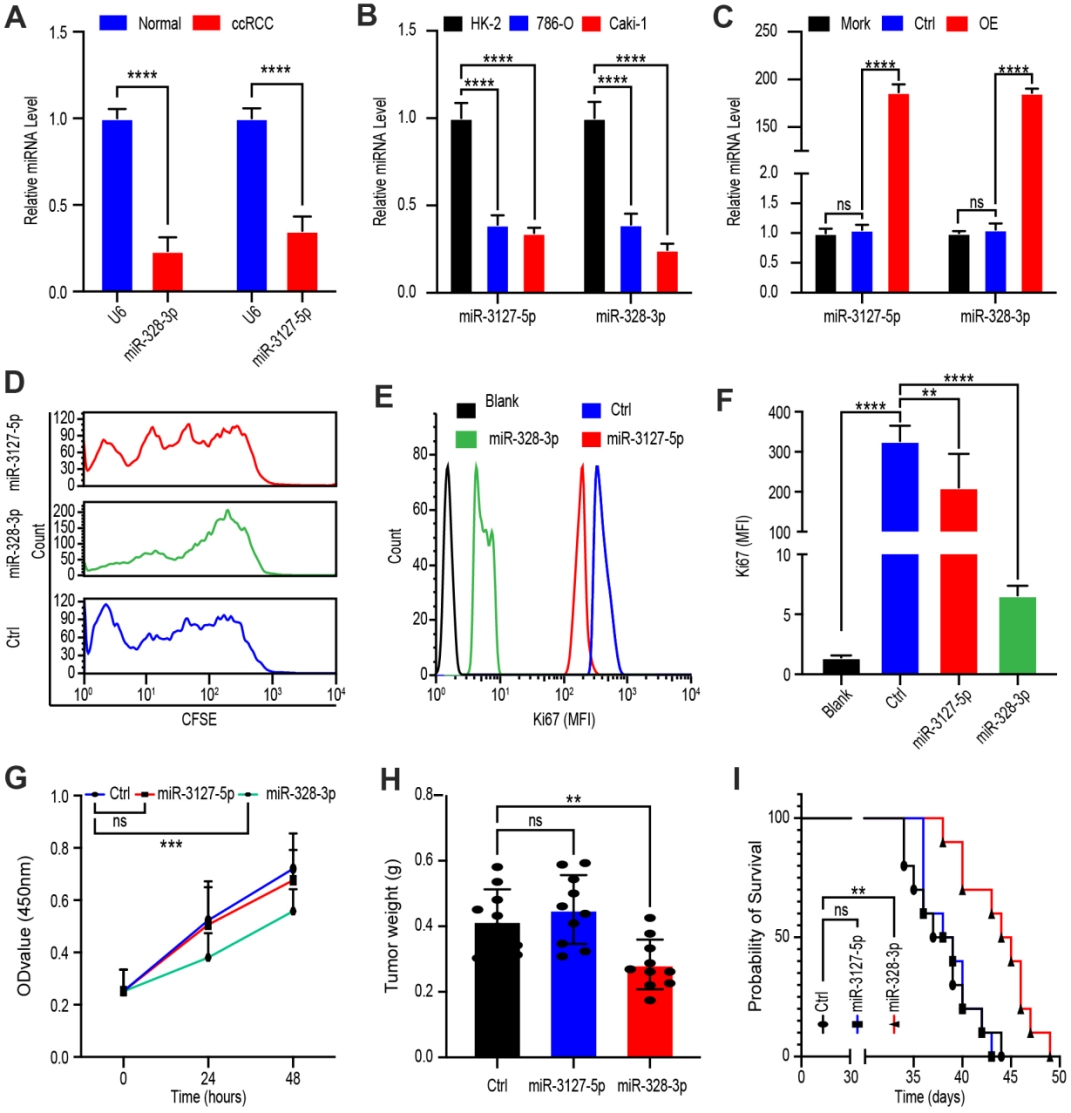
**PVT1 promotes ccRCC proliferation mainly through miR-328-3p.** (**A**) qRT-PCR assays of miR-328-3p and miR-3127-5p in ccRCC tissues and adjacent normal renal tissues (n=45). (**B**) Expression levels of miR-328-3p and miR-3127-5p in ccRCC cell lines, 786-O, and Caki-1 compared with those in the normal renal cell line, HK-2, were determined by qRT-PCR. (**C**) Caki-1 cells treated with Mork, Lentivirus vector, OE-miR-328-3p lentivirus, or OE-miR-3127-5p lentivirus for 24 hours; levels of miR-328-3p and miR-3127-5p were determined by qRT-PCR. (**D**) Fluorescence attenuation of CFSE-labeled Caki-1 cells after treatment with lentivirus vector, OE-miR-328-3p lentivirus, or OE-miR-3127-5p lentivirus for 48 hours. (**E**, **F**) Flow cytometry assays to determine MFI of Ki67 in Caki-1 cells treated with empty lentivirus vector, OE-miR-328-3p lentivirus, or OE-miR-3127-5p lentivirus for 48 hours. (**G**) Cell viability was determined by CCK-8 assays after treatment with lentivirus vector, OE-miR-328-3p lentivirus, or OE-miR-3127-5p lentivirus; data were recorded every 24 hours. (**H**) Tumor weight of ccRCC xenograft models harboring tumors generated by cells treated with lentivirus vector OE-miR-328-3p lentivirus or OE-miR-3127-5p lentivirus for 30 days. (**I**) Survival time of ccRCC xenograft models. Mean ± SEM, *P < 0.05, ** P < 0.01, ****P < 0.001, NS: no significance.

To determine whether miR-328-3p and/or miR-3127-5p are the target miRNAs of PVT1 for its regulation of tumor cell proliferation, we investigated the levels of miR-328-3p as well as miR-3127-5p in tissues of ccRCC and adjacent normal parts from 45 patients. qRT-PCR analysis elucidated that both miR-3127-5p and miR-328-3p were downregulated in ccRCC tissue (Figure 4A). Moreover, comparing to that in the normal kidney cell line, HK-2, miR-328-3p and miR-3127-5p were down-regulated in 786-O and Caki-1 which are cell lines of ccRCC (Figure 4B).

Next, to exploit the potential role of miR-3127-5p and miR-328-3p, Caki-1 cells were treated with Mock, lentivirus vector, OE-miR-3127-5p lentivirus, or OE-miR-328-3p lentivirus for 24 hours. qRT-PCR assays confirmed that OE-miR-3127-5p and OE-miR-328-3p lentiviruses significantly increased miRNA levels (Figure 4C). Next, lentivirus-treated cells were labeled with CFSE for 48 hours, after which flow cytometry analysis revealed rapid fluorescence attenuation in OE-miR-328-3p lentivirus-treated cells, whereas no obvious attenuation was observed in OE-miR-3127-5p lentivirus-treated cells (Figure 4D).

Since Ki67 is the protein marker of proliferation, we determined its MFI following treatment with lentivirus vector, OE-miR-3127-5p lentivirus or OE-miR-328-3p lentivirus for 48 hours and discovered that OE-miR-328-3p lentivirus treatment statistically cut down the MFI of Ki67 (Figure 4E, 4F). To confirm this phenomenon, we determined the viability of Caki-1 cells every 24 hours after treatment with the abovementioned vectors via CCK-8 assays and found that miR-328-3p overexpression statistically hindered proliferation (Figure 4G).

An *in vivo* ccRCC xenograft mouse model was then established via subcutaneous injection of Caki-1 cells treated with lentivirus vector, OE-miR-3127-5p lentivirus, or OE-miR-328-3p lentivirus. Results showed that tumor weight was decreased in the OE-miR-328-3p lentivirus-treated group but not in the OE-miR-3127-5p lentivirus-treated group (Figure 4H). Survival time of OE-miR-328-3p lentivirus-treated group was shorter but not in the OE-miR-3127-5p lentivirus-treated group (Figure 4I). Cumulatively, these consequences elucidated that miR-328-3p is the primary target miRNA of PVT1 in the modification of the proliferation in ccRCC cells.

### FAM193B is the target gene of miR-328-3p

**Figure 5:**
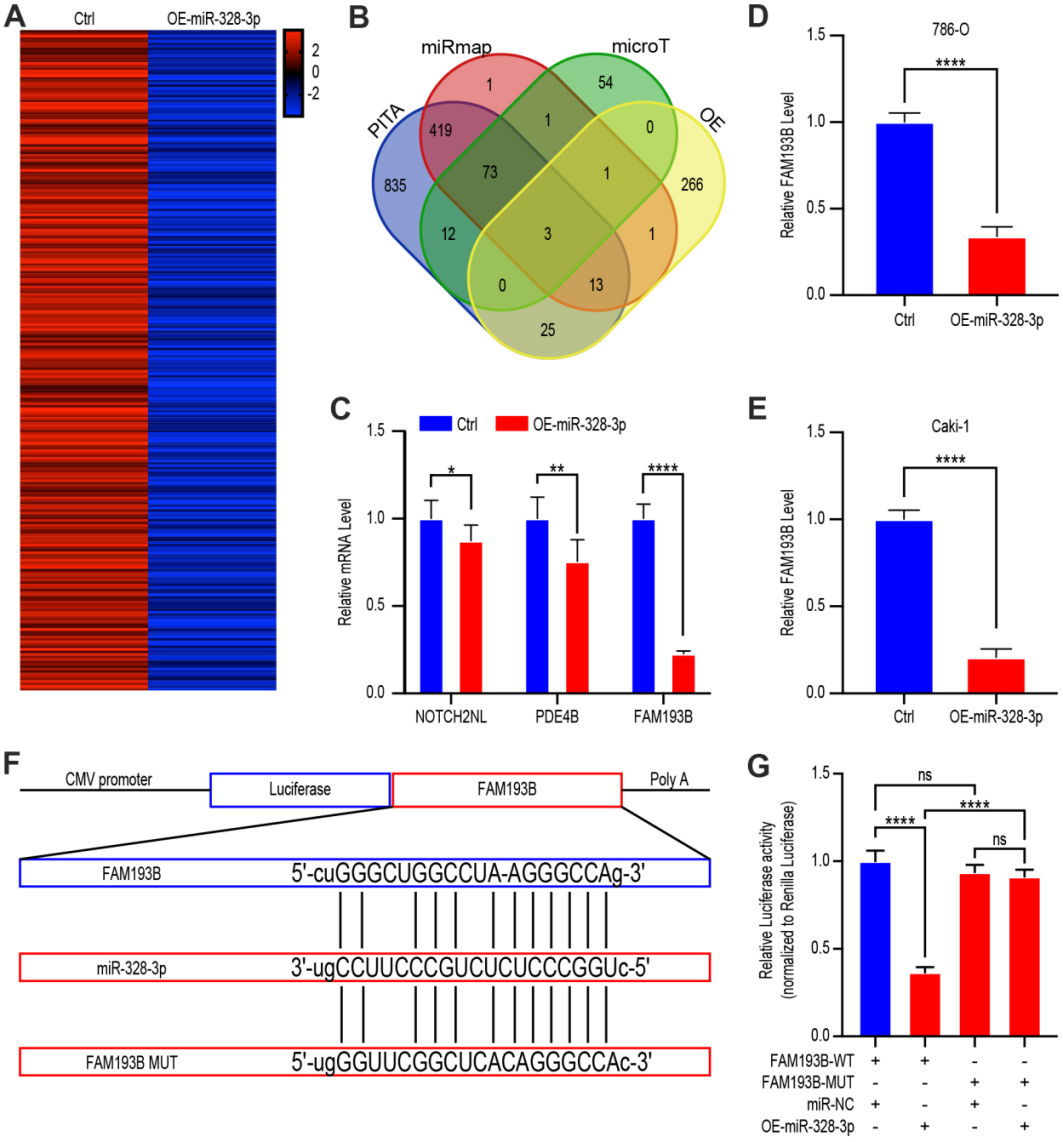
**FAM193B is a miR-328-3p target gene by binding to 3’UTR site.** (**A**) Heat map of down-regulated genes in Caki 1 cells treated with OE-miR-328-3p lentivirus compared with cells treated with lentivirus vector (fold change > 2.0, P < 0.05). (**B**) Venn diagram showing the over-lapping of potential target genes of miR-328-3p in the results of gene chip assay and bioinformatic analysis of PITA, miRmap, and microT. (**C**) qRT-PCR assays of potential target genes, NOTCH2NL, PDE4B, and FAM193B, in 293T cells treated with lentivirus vector or OE-miR-328-3p lentivirus. (**D**, **E**) 786-O and Caki 1 cells treated with lentivirus vector or OE-miR-328-3p lentivirus for 24 hours. FAM193B levels were determined by qRT-PCR. (**F**) Schematic view of putative miR-328-3p targets in the WT and MUT 3’UTR of FAM193B. (**G**) Luciferase activity assay of 293 T cells transfected with luciferase reporter plasmids harboring FAM193B 3’UTR (WT or MUT) and control miRNA or miR-328-3p. Mean ± SEM, ** P < 0.01, ***P < 0.001, ****P < 0.001, NS: no significance.

Next, we sought to identify the target site of relevant of miR-328-3p. We, therefore, treated Caki-1 cells with lentivirus empty vector or OE-miR-328-3p lentivirus and used gene chip analysis to determine the latent target sites, which would appear as significantly down-regulated after treating with the OE-mir-328-3p lentivirus (P<0.05, fold change>2.0) (Figure 5A). We also predicted the miR-328-3p target genes using miRmap (http://mirmap.ezlab.org/), PITA (http://genie.weizmann.ac.il/pubs/mir07/mir07_data.html), and microT (http://www.microrna.gr/microT) databases, as well as analysis of the gene expression in miR-328-3-upregulated Caki-1 cells. The over-lapping potential target genes identified by these analyses were NOTCH2NL, PDE4B, and FAM193B (Figure 5B). To verify this predicted result, qRT-PCR of the three potential genes was conducted and found that FAM193B was decreased more significantly than the other genes in 293T cells transfected with OE-miR-328-3p lentivirus for 24 hours (Figure 5C). Similarly, in malignant 786-O and Caki-1, FAM193B decreased following OE-miR-328-3p lentivirus transfection (Figure 5D, 5E). Hence, FAM193B was sifted for further exploitation in ccRCC cells.

Analysis *in silico* unveiled that the 3′ UTR of FAM193B contains a potential miR-328-3p–binding site (Figure 5F). We, therefore, performed luciferase reporter assays using a construct containing the luciferase gene actuated by the sequence of wild-type 3′ UTR of FAM193B, containing the miR-328-3p–binding site (FAM193B-WT) of prediction, or with structures including a miR-328-3p–binding sites mutation (FAM193B-MUT). Co-transfection into 293T cells of these plasmids were performed together with OE-miR-328-3p plasmids or nontargeting control miRNA. The consequences elucidated that WT-FAM193B–actuated luciferase activity was statistically decreased by co-transfecting with the OE-miR-328-3p plasmids comparing to the control, however, this suppression was hindered by the putative miR-328-3p–binding site mutation in the FAM193B 3′UTR (Figure 5G). Cumulatively, these consequences depicted that miR-328-3p modifies expression of FAM193B in ccRCC cells by binding straightly to the putative site in the 3′UTR of FAM193B mRNA.

### FAM193B as the target gene of miR-328-3p is indirectly modulated by PVT1

**Figure 6:**
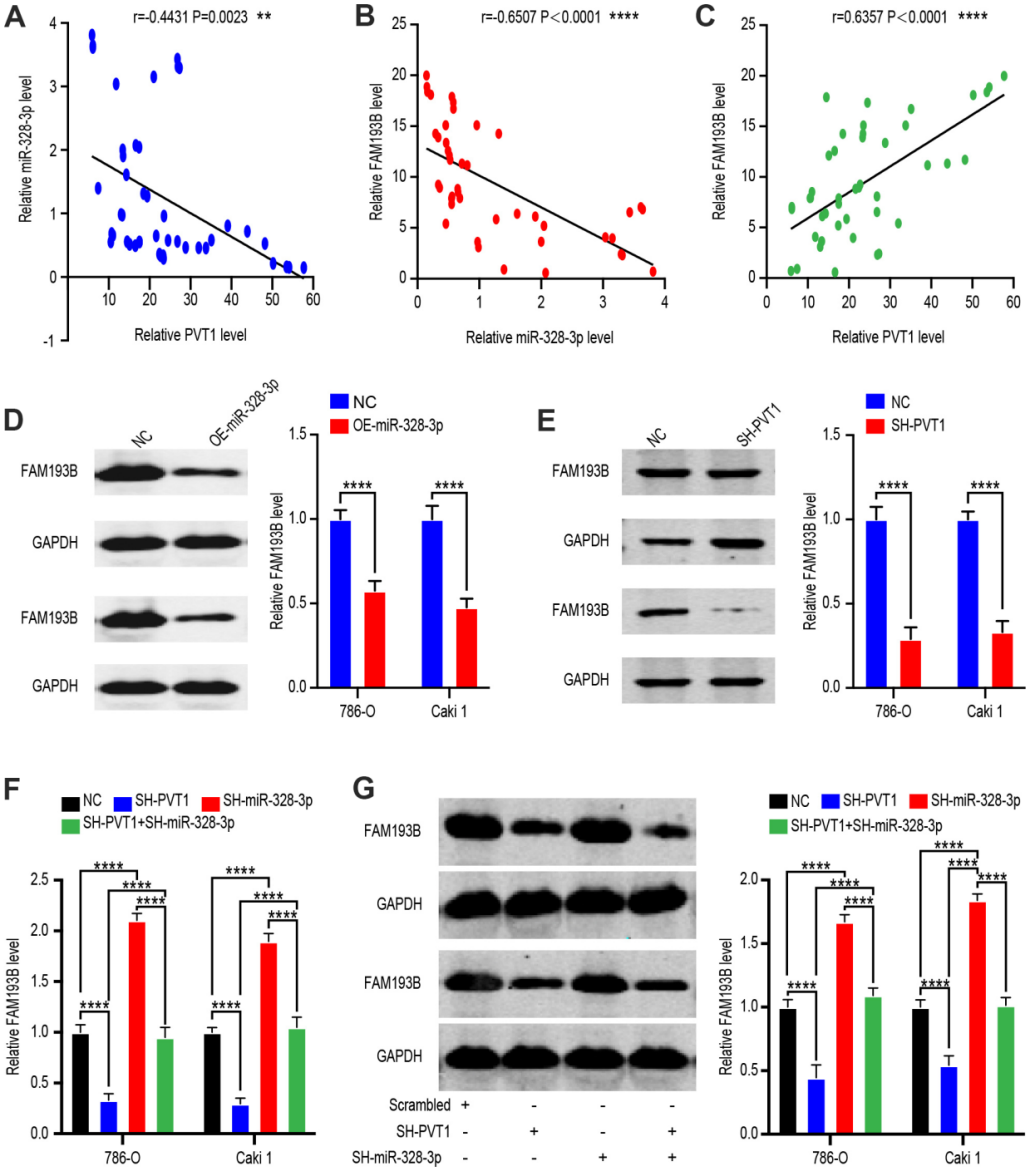
**FAM193B is a target of miR-328-3p and is suppressed by PVT1 deletion.** (**A**–**C**) Correlation between PVT1-miR-328-3p expression levels (**A**), miR-328-3p-FAM193B expression levels (**B**) and PVT1-FAM193B expression levels (**C**) in 45 paired ccRCC tissues. (**D**) Relative protein levels of FAM193B in DU 145 cells transfected with empty lentivirus vector and OE-miR-328-3p Lentivirus. (**E**) Relative protein levels of FAM193B in DU 145 cells transfected with empty lentivirus vector and SH-PVT1 Lentivirus. (**F**) FAM193B mRNA and protein levels in 786-O and Caki-1 cells following knockdown of PVT1 and/or inhibition of miR-328-3p. (**G**) Protein level in 786-O and Caki-1 cells following knockdown of PVT1 and/or inhibition of miR-328-3p. Mean ± SEM, ** P < 0.01, ****P < 0.001, NS: no significance.

To identify the network of ceRNA between PVT1, miR-328-3p, and its target gene, FAM193B, in ccRCC, we collected 45 ccRCC tissues and quantified the RNA levels of PVT1 and FAM193B relative to that of GAPDH and RNA levels of miR-328-3p relative to that of U6. Negative correlations were observed between miR-328-3p and PVT1, and between FAM193B and miR-328-3p, while a positive correlation was noted between PVT1 and FAM193B. These results were concordant with the possibility of a regulatory PVT1–miR-328-3p–FAM193B axis (Figure 6A–6C). To confirm these results, we quantified FAM193B protein levels in Caki-1 and 786-O cells with miR-328-3p overexpression or PVT1 suppression (Figure 6D, 6E). To determine whether miR-328-3p contributes to the association between PVT1 and FAM193B, we examined cells of co-transfection with the SH-miR-328-3p and SH-PVT1 lentivirus. Indeed, the repression of FAM193B protein and mRNA levels induced by SH-PVT1 lentivirus was efficiently conversed by the SH-miR-328-3p lentivirus (Figure 6F, 6G). Conclusively, these consequences demonstrated that PVT1 regulates the FAM193B expression via regulation of miR-328-3p of posttranscription.

### FAM193B promotes proliferation by upregulating the PI3K/AKT and MAPK/ERK signaling pathways

**Figure 7:**
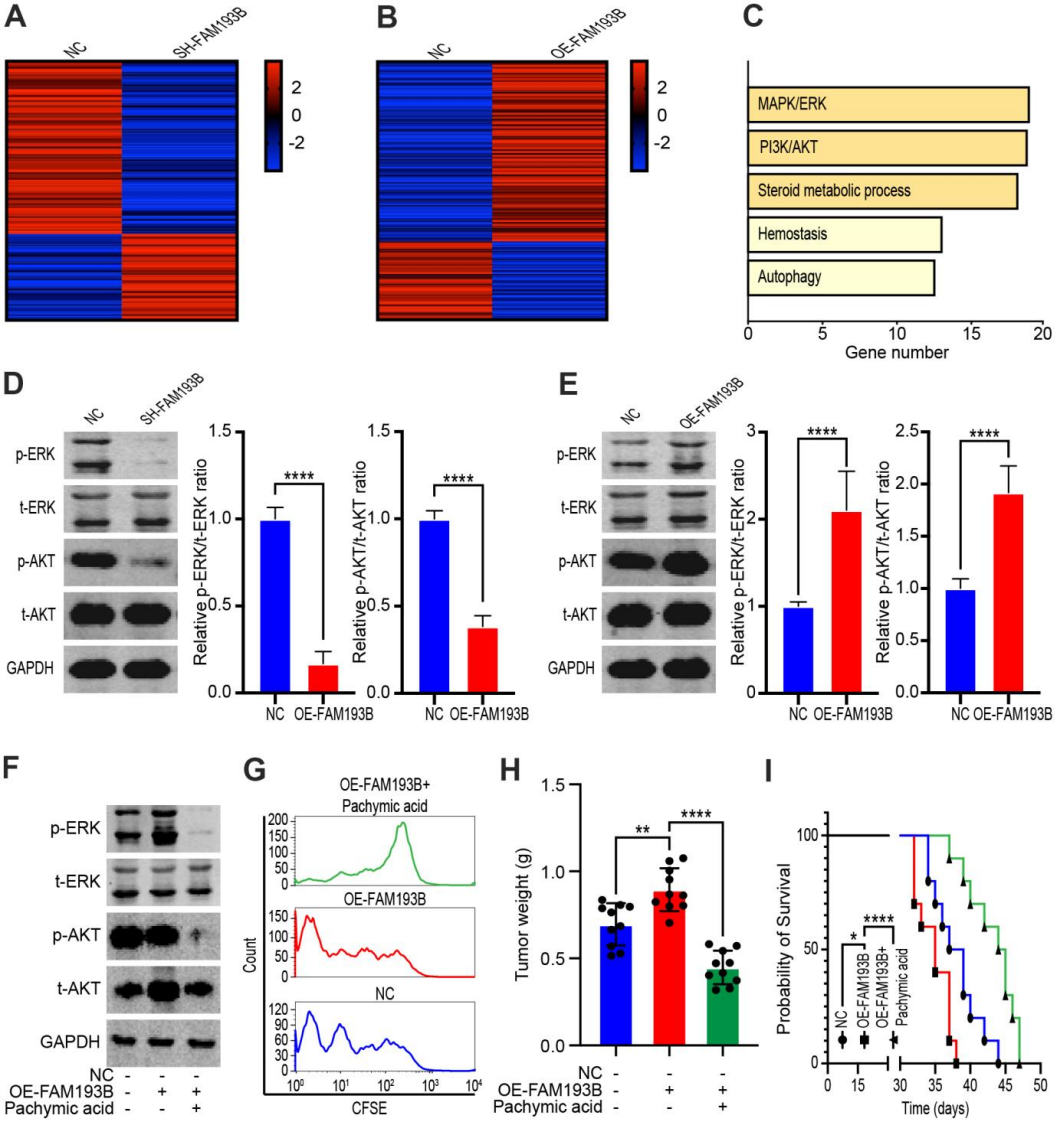
**FAM193B promoted ccRCC proliferation by activating the MAPK/ERK and PI3K/AKT pathways.** (**A**, **B**) Heatmap of differentially expressed genes in DU 145 transfected with SH-FAM193B lentivirus (**A**) or OE-FAM193B lentivirus (**B**) compared with empty lentivirus vector-transfected cells. (**C**) Gene enrichment analysis of differentially expressed genes regulated by FAM193B. D, E. The Caki-1 cells were treated with SH-FAM193B lentivirus (**D**) or OE-FAM193B lentivirus (**E**), and cell lysates were then subjected to western blot analysis to determine the levels of phosphor-ERK (p-ERK), total ERK (t-ERK), phosphor-AKT (p-AKT), and total AKT (t-AKT) compared with those in cells treated with empty lentivirus vector. (**F**) The levels of phosphor-ERK (p-ERK), total ERK (t-ERK), phosphor-AKT (p-AKT), and total AKT (t-AKT) in Caki-1 cells treated with OE-FAM193B lentivirus and/or PA compared with those in empty lentivirus vector-treated cells. (**G**) Fluorescence attenuation in CFSE-labeled-Caki-1 cells following OE-FAM193B lentivirus and/or PA treatment for 48 hours compared with that in empty lentivirus vector-treated cells. (**H**) Tumor weight of ccRCC xenograft models harboring tumors generated by cells treated with OE-FAM193B lentivirus and/or PA for 30 days compared with those in mice harboring tumors generated by empty lentivirus vector-transfected cells. (**I**) Survival time of ccRCC xenograft models. Mean ± SEM, ****P < 0.001.

To explore the mechanism through which FAM193B promotes the proliferation of ccRCC cells, we transfected Caki-1 cells with lentivirus empty vector, SH-FAM193B lentivirus, or OE-FAM193B lentivirus and used gene chips to detect genes displaying significant differential expression (Figure 7A, 7B). Results from the gene enrichment analysis identified differentially expressed genes enriched in several pathways, with five signaling pathways exhibited obvious activation (Figure 7C). Specifically, the PI3K/AKT and MAPK/ERK signaling pathways perform a momentous role in ccRCC cancerogenesis and proliferation. We, thus, managed to dig the effect of FAM193B on both signaling pathways in ccRCC cells. SH-FAM193B lentivirus treatment markedly inhibited MAPK/ERK axis in ccRCC cells, evidenced by the phosphorylation reduction of ERK in Caki-1 and 786-O cells. Moreover, we uncovered that SH-FAM193B lentivirus intervention reduced the AKT phosphorylation and total AKT levels in ccRCC cells, consequently hindered the activity of PI3K/AKT axis (Figure 7D). To verify this phenomenon, we repeated these experiments by transfecting cells with OE-FAM193B lentivirus, and found that phosphorylation of both ERK and AKT increased with OE-FAM193B lentivirus treatment (Figure 7E). PA, a lanostane-type triterpenoid, inhibits the AKT and ERK signaling pathways [13]. Hence, to confirm whether FAM193B promotes ccRCC proliferation via the PI3K/AKT and MAPK/ERK signaling pathways, we treated Caki-1 cells with OE-FAM193B lentivirus or PA. PA treatment significantly decreased OE-FAM193B-mediated protein phosphorylation of ERK and AKT (Figure 7F). Furthermore, flow cytometry of Caki-1 cells labeled with CFSE for 48 hours revealed rapid fluorescence attenuation in OE-FAM193B lentivirus-treated cells, whereas PA treatment reversed this phenomenon (Figure 7G). *In vivo*, Caki-1 cell line were performed stable transfection with OE-FAM193B lentivirus or control vector and engrafted subcutaneously into immunodeficient nude mice. Oral administration of PA significantly inhibited the tumor-promoting effect of OE-FAM193B lentivirus (Figure 7H, 7I). Thus, our data indicates that FAM193B promotes proliferation by upregulating the PI3K/AKT and MAPK/ERK signaling pathways and that this effect can be reversed by PA.

### Clinic value of PVT1 united with FAM193B in several cancers

**Figure 8:**
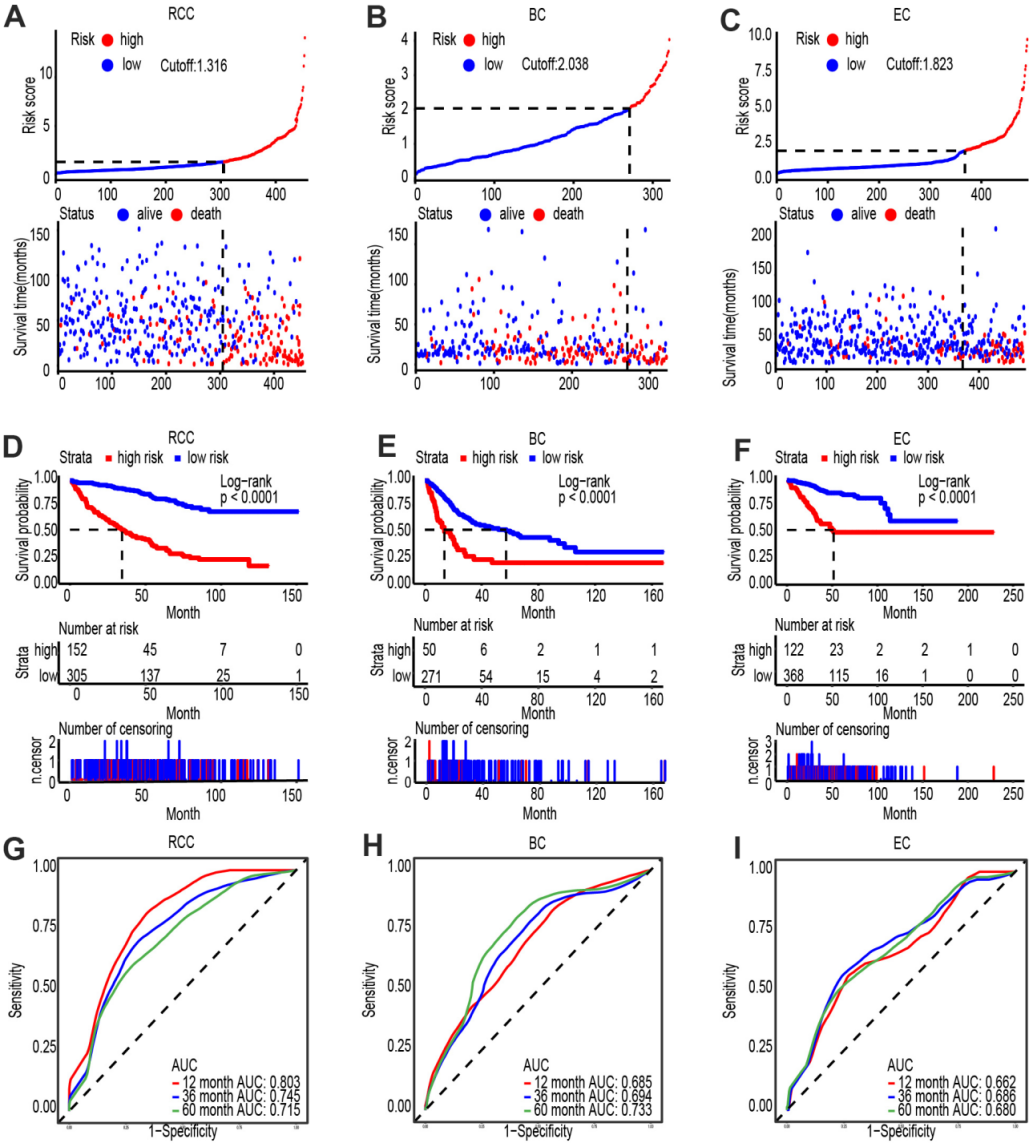
**PVT1 and FAM193B are highly expressed and associated with poor outcome in several cancers.** (**A**–**C**) Risk score analysis of Shanghai General Hospital ccRCC (**A**), BC (**B**) and EC (**C**) patient cohorts. The risk score and patient distribution were visualized with the best cut-off value. (**D**–**F**). Patients were divided into low-risk and high-risk groups based on the optimal cut-off point. Kaplan-Meier method was applied to estimate the survival status. (**G**–**I**). To compare the sensitivity and specificity of survival predication, ROC analysis of 3- and 5-year OS was performed based on the risk score.

Accumulating evidence shows that lncRNAs and mRNAs are promising biomarkers for the diagnosis and prognosis of ccRCC, respectively. Thus, identifying the clinical value as well as significance of PVT1 and FAM193B, especially of their combined effect, in ccRCC would possess tremendous clinical value. Through the risk evaluation established by multivariate Cox regression model, we stratified ccRCC, bladder cancer (BC) and endometrial cancer (EC) patients into high and low PVT1-FAM193B groups (Figure 8A–8C). Based on the follow up data for ccRCC, BC, and EC, patients with high PVT1-FAM193B levels were found to have lower survival times than patients with low PVT1-FAM193B levels (Figure 8D–8F). Further, we determined the cut-off value of premium for the stratification of two risk groups and a survival outcome of significant differentiation was obtained (Log-rank P < 0.0001). Receiver operator characteristic (ROC) curve of 3- and 5-year overall survival (OS) was implemented and the area under the curves (AUCs) were calculated, respectively (Figure 8G–8I).

## DISCUSSION

Categorized as non-coding transcripts, lncRNAs are broadly investigated for their potential role in disease pathogenesis [14]. Moreover, the majority of literature focuses on their role in tumor proliferation, distant metastasis, and chemo-resistance [15–17]. In this study, using high-throughput analysis, we identified that lncRNA PVT1 is aberrantly expressed in ccRCC and is associated with unfavorable OS. Furthermore, knockdown of PVT1 could effectively inhibit malignant cell invasion, migration, and proliferation, concordant with previous researches in other malignancies.

A broader perspective has recently been adopted by researchers that lncRNA harbors an miRNA-response structure that acts as a decoy and prevents miRNAs from integrating with protein-coding RNAs; such lncRNAs are now recognized to be part of a ceRNA network [18]. Functional crosstalk in the ceRNA framework can assist in coordinating various biological pathways, while their impairment could result in disease development [19]. In this study, we revealed that PVT1 interacts with FAM193B by binding to miR-328-3p, which leads to the activation of ERK signaling, thereby promoting RCC cell invasion and disease progression.

In a previous investigation, miR-328-3p was described as an inhibitor of cancer cell proliferation. Luo reported that miR-328-3p could inhibit breast cancer cell proliferation by targeting receptor for advanced glycosylation end products (RAGE) [20]. Similarly, Zhang and Li provided evidence that LINC00210-mediated silencing of the miR-328-3p/NOTCH3 pathway facilitated nasopharyngeal carcinoma development and progression [21]. These findings are consistent with our results that miR-328-3p acts as a neoplasm repressor in RCC cells.

Through bioinformatic analysis, we identified FAM193B as a candidate target for miR-328-3p. FAM193B, also known as IRIZIO, has been found to cooperate with PAX3-FOXO1 fusion genes in alveolar rhabdomyosarcoma tumorigenesis [22]. Here, we proved that increased levels of FAM193B facilitates RCC cell proliferation by activating the AKT and ERK pathway.

Finally, we investigated the potential of PVT1, FAM193B, and miR-328-3p expression to predict patient survival. When used in combination with clinical stage and age of patients, we are able to stratify the patients into different risk levels based on PVT1, FAM193B, and miR-328-3p expression. ROC analysis also supported their ability to predict patient OS. Thus, these potential novel markers could help stratify patients for determining optimal treatment and pave the way toward personalized medicine to offer improved therapeutic benefits.

## CONCLUSIONS

We elucidated the potential feedback loop link between PVT1/miR-328-3p/FAM193B, which reflects the significant role of PVT1 in RCC. Our conclusions also imply that the ceRNA framework may be a practical prognostic biomarker, and targeting the PVT1/miR-328-3p/FAM193B axis, might represent a rational therapeutic strategy for RCC.

## MATERIALS AND METHODS

### Tissue samples

We acquired 45 coupled ccRCC tissues and adjacent normal parts based on histopathologic evaluation by laser capture microdissection (LCM) at the Shanghai General Hospital between 2015 and 2018. All ccRCC sufferers did not undergo radiotherapy or chemotherapy prior to surgery. In accordance with the International Union against Cancer/American Joint Committee on Cancer system, the tumor–node–metastasis (TNM) staging system was utilized to stage histologic grade. All research and experiments abided by the principles of the Declaration of Helsinki. All ccRCC sufferers’ clinical characteristics were summarized and recorded in Table 1. The informed consent was obtained and the research was approved by the Research Ethics Committee of Shanghai Jiaotong University.

**Table 1:** Clinical characteristics of ccRCC patients.

**Characteristics**	**Groups**	**No.**	**PVT1**	**P-value**	**miR-328-3p**	**P-value**	**FAM193B**	**P-value**
Tissue	ccRCC	45	23.48±2.02	<0.0001	1.25±0.17	<0.0001	9.36±0.81	<0.0001
	nontumor	45	1.217±0.07		3.53±0.07		1.025±0.04	
Age, years	≤60	11	20.95±3.66	0.4821	1.51±0.38	0.3919	10.05±1.53	0.6360
	>60	34	24.30±2.41		1.17±0.19		9.14±0.97	
Gender	Male	30	23.58±2.47	0.9434	1.27±0.22	0.8021	10.14±0.95	0.1803
	Female	15	23.27±3.63		1.19±0.28		7.81±1.49	
TNM stage	I/II	35	20.63±1.95	0.0068	1.40±0.20	0.0879	8.09±0.84	0.0024
	III/IV	10	33.46±5.04		0.71±0.27		13.82±1.55	

### RNA collection and quantitative reverse transcription polymerase chain reaction (qRT-PCR)

RNA of totality, including lncRNAs, miRNAs and mRNAs were collected utilizing the kit of RNAsimple Total RNA (DP419, Tiangen Biotech, China) and reverse transcription (RT) of total RNA was implemented utilizing the kit of FastKing RT (KR116, Tiangen Biotech). RT of miRNA was implemented utilizing the kit of miRcute Plus miRNA First-Strand cDNA Synthesis (KR211, Tiangen Biotech) and lncRNAs/mRNA using TIANScript II RT Kit (KR107, Tiangen Biotech). All samples were evaluated utilizing the kit of Quant One Step RT-qPCR (SYBR-Green) (FP304, Tiangen Biotech). The conditions of thermocycling were performed according to manuscripts. GAPDH and U6 were utilized for normalization, and the lncRNAs, miRNAs and mRNAs’ levels of relative expression were analyzed utilizing the 2-ΔΔCT method (Livak KJ and Schmittgen TD, Methods 25: 402- 408, 2001.). Primers used were synthesized by Tiangen Biotech (China) (Table 2).

**Table 2:** Primers for qRT-PCR.

**Name**	**Forward primers 5′-3′**	**Reverse primers 5′-3′**
PVT1	CCTGTGACCTGTGGAGACAC	GTCCGTCCAGAGTGCTGAAA
FAM193B	AGTGCAGAAACCCCACCAAA	ATGACGAACCCAACCTGGTG
miR-3127-5p	GGCCCATCAGGGCTTGTG	GCCACACCCAGCAGGC
miR-328-3p	CGGGCCTGGCCCTCTCTGCC	CAGCCACAAAAGAGCACAAT
NOTCH2NL	CTAGCACAGAAGCCCATCCC	GGGAATGGGGGAATCAACAGT
PDE4B	TGGCCTCAGCAGTTACAACC	CTCCTAGGCAGCCCTTACCT
U6	ATTGGAACGATACAGAGAAGATT	GGAACGCTTCACGAATTTG
GAPDH	AGACAGCCGCATCTTCTTGT	TGATGGCAACAATGTCCACT

### Western blot protocol and analysis

All cells after treatment were gathered and lysates were treated in lysis buffer (P0013K, Beyotime Institute of Biotechnology, China) containing a cocktail of protease inhibitor (P1050, Beyotime Institute of Biotechnology). Each lysate of total cell (30 μg/lane) was divided on 12% SDS-PAGE gels and electronically transferred to membranes of PVDF (ISEQ00010, Millipore, USA). In the wake of antigen blockading with QuickBlock™ Blocking Buffer (P0252; Beyotime Institute of Biotechnology), the PVDF membranes were implemented with anti-FAM193B (1-2 μg/ml, ab139820, Abcam, USA), anti-p-ERK (1/500, ab65142, Abcam), anti-t-ERK (1/1000, ab17942, Abcam), anti-p-AKT: (1/500, ab8805, Abcam), anti-t-AKT (1/10000, ab179463, Abcam) overnight. Subsequently, the membranes washed with TBST, were implemented with the secondary antibody (1/2000, ab205718, Abcam) and visualized with SuperSignal West Femto maximum sensitivity substrate (34095, Thermo Fisher, USA) by Image Lab software. The experiments were implemented in triplicate.

### Cell culture and cell viability assay

CcRCC cell lines of human, Caki-1 and 786-O, and normal renal cell line of human, HK-2 were provided by the Typical Culture Preservation Commission Cell Bank of the Chinese Academy of Sciences (China). The cell culture medium was RPMI-1640 (31800022, Gibco, USA) with 10% fetal bovine serum (SH30084.03, HyClone, USA) under an atmosphere of humidification with CO2 of 5% and the temperature of cell culture was set at 37° C. Treated cells (3000/well) were seeded in 96-well plates. CCK-8 assay (GK10001, Glpbio, USA) was utilized to check the relative growth of treated or control cells every 24 hours in line with the protocols from manufacturer.

### Viral transduction

The GFP-labeled lentivirus vectors including the OE-miR-328-3p lentivirus, OE-miR-3127-5p lentivirus, OE-PVT1 lentivirus, OE-FAM193B lentivirus and short hairpin RNA (shRNA) were used to overexpress target genes and SH-miR-328-3p lentivirus, SH-PVT1 lentivirus and SH-FAM193B lentivirus were used to silence target genes. All these lentiviruses were acquired from GeneChem (China) and before transduction, all cells were inoculated in 6-well plates (5×105 cells/well). Lentiviral vectors transduction was processed utilizing 8 mg/ml Polybrene (Genechem) and transduction reagents for 12 h. The multiplicity of infection (MOI) was 10,100 or 1,000 when transducing cells during viral transduction. Silencing, overexpression and the relative control stable cells were subsequently developed, and the transduction efficiency was verified utilizing the way of RT-qPCR. SH-PVT1 sequences targeting is CCUGAUGGAUUUACAGUGATT. SH-FAM193B sequences targeting is GCTCAAAGGAAGTTCCCAGTT.

### Luciferase analysis

Downstream of the luciferase gene within the pGL3-Baisc luciferase reporter vector (GeneChem, China), the fragment of complementary DNA comprising the mutant or wild-type PVT1 fragment and 3’ untranslated region (UTR) of FAM193B was subcloned. 293T cell line of human (1.0×10^5^) seeded in a 24-well plate were implemented co-transfection with 150 ng of either miR-3127-5p and miR-328-3p or empty vector, firefly luciferase reporter of 50 ng containing mutant or wild-type PVT1 and 3’ UTR of FAM193B utilizing Lipofectamine 3000 (L3000-015, Invitrogen, USA). Luciferase analysis was assessed utilizing the DualLuciferase Kit (E1910, Promega, USA) 48 hours after transfection. To those of Renilla luciferase the relative activities of firefly luciferase were normalized. The transfection assay was echoed in triplicate.

### RNA immunoprecipitation

Immunoprecipitation of RNA was utilized to exploit whether PVT1 could bind with the latent binding target Ago2. The Kit of EZMagna RIP (17-701, Millipore) in line with the protocol of manufacturer. Cells were totally lysed and implemented with magnetic beads of protein A, conjugated with antibodies, at 4° C. Counting 5 hours, the magnetic beads were rinsed with buffer and then implemented with SDS/0.5 mg/mL Proteinase K of 0.1% for half an hour at 55° C to eliminate proteins. Eventually, RNA of immunoprecipitation was sent to qRT-PCR assay to illustrate the existence of PVT1 utilizing primers of specificity (Table 2).

### Flow cytometric assay

Cells being harvested which were subject to transfection with OE-PVT1 lentivirus, SH-PVT1 lentivirus, OE-miR-328-3p lentivirus, OE-miR-3127-5p lentivirus, OE-FAM193B lentivirus or empty vector lentivirus for 48 hours. After staining with CFSE (65-0850-84, Thermo Fisher), the targeted cells were evaluated by a flow cytometer (FACScan; BD Biosciences, USA) with the installation of the software of CellQuest (BD Biosciences). Ki67 as a protein of nuclear is of necessity for cell proliferation [23]. All cells, thus, were incubated with anti-Ki67 antibodies (1:20; 350514; BioLegend, USA) for half an hour, and the IgG antibody (1:20; 400141; BioLegend) was utilized as a control. The mean fluorescence intensity (MFI) was checked with the assay of CBA (BD Biosciences) and the experiments were performed in triplicate.

### Animal experiments

To develop a mouse model of xenograft, nude mice of 4 weeks old from SLAC Laboratory Animal Center (China) were used, and 5×10^6^ Caki-1 cells were subcutaneously injected into the region of nude mice armpit. Mice weights were determined every 7 days. To evaluate the *in vivo* effect of pachymic acid (PA), the mice were implemented with PA (60 mg/kg) through injections in intraperitoneal (ip) way for 5 days per week until 3 weeks. The survival time of mice were recorded. the experiments of animal were approved by the Institutional Review Board of Shanghai Jiaotong University.

### Statistical analysis

Data are illustrated as mean ± SEM unless manifest otherwise. The difference of statistical significance between the experiment groups and control was identified by either simple one-way ANOVA following Tukey’s post hoc test for multiple comparisons or Student t test utilizing Prism 7.0 (GraphPad Software, USA). P < 0.05 were deemed statistically significant.
